# Sociodemographic and clinical characteristics of the population with a first psychotic episode attended in the mental health services of area 5 of Madrid (Spain)

**DOI:** 10.1192/j.eurpsy.2023.953

**Published:** 2023-07-19

**Authors:** J. Garde González, P. Herrero Ortega, A. Oliva Lozano, I. I. Louzao Rojas, M. P. Vidal-Villegas, A. Muñoz-Sanjosé, M. P. Sánchez-Castro, G. Lahera, S. Sánchez Quílez, M. F. Bravo-Ortiz, O. B. O. A.-M. Group

**Affiliations:** 1 Department of Psychiatry, Clinical Psychology and Mental Health, La Paz University Hospital; 2 Department of Psychiatry, Autonomous University of Madrid (UAM); 3 Hospital La Paz Institute for Health Research (IdiPAZ); 4 Department of Psychiatry and Mental Health, University of Alcalá; 5 Centro de Investigación Biomédica en Red de Salud Mental (CIBERSAM), Madrid, Spain

## Abstract

**Introduction:**

Risk of functional impairment and progression to chronic illness in people with a first episode of psychosis (FEP) has motivated early intervention programs, showing promising results. Defining the characteristics of people with FEP at local level enables the clinicians to adjust interventional models to the reality of the population. The area 5 of Madrid (Spain) is referred to La Paz University Hospital and it serves a catchment area of roughly 527,000 people.

**Objectives:**

We aim to identify sociodemographic and clinical characteristics of patients in the area 5 of Madrid (Spain) who meet the criteria of FEP.

**Methods:**

A descriptive retrospective study including 179 people (age range 18-40 years) who were attended in mental health services of La Paz University Hospital (area 5 of Madrid, Spain), between January 2019 and May 2020, having suffered a psychotic episode in the last five years.

**Results:**

The average age of people with FEP was 29.32 years, with a higher proportion of men (62%). The mean duration of untreated psychosis (DUP) was 3.64 months and 47% of patients consume cannabis. We found disparities in DUP among the different districts in the area and we also observed differences depending on the district for inclusion in rehabilitation programs or psychotherapy. The following averages were obtained for the aggregate sample: 1.01 hospitalization/year, 1.42 emergency room visits/year, 1.81 years of illness and a mean dosage equivalent to olanzapine 6.75 mg/day. The incidence of psychosis in our area has been 7.01 cases per 100000 inhabitants/year.

**Conclusions:**

The incidence of psychosis has been as expected according to data recorded at previous studies in Spain. The results obtained in our sample have included a lower DUP and a higher use of cannabis than those described in the literature. We have also found differences when observing the inclusion of patients in different treatments (psychotherapy, rehabilitation), which may be related to the differences in the DUP by districts. Further exploration in this field is needed to draw causal conclusions.

**Disclosure of Interest:**

None Declared

